# Review of Research into the Determination of Acrylamide in Foods

**DOI:** 10.3390/foods9040524

**Published:** 2020-04-22

**Authors:** Mingfei Pan, Kaixin Liu, Jingying Yang, Liping Hong, Xiaoqian Xie, Shuo Wang

**Affiliations:** 1 State Key Laboratory of Food Nutrition and Safety, Tianjin University of Science & Technology, Tianjin 300457, China; panmf2012@tust.edu.cn (M.P.); lkx13642168374@163.com (K.L.); yangjy0823@126.com (J.Y.); honglpstu@163.com (L.H.); xiexiaoqian8135@163.com (X.X.); 2 Key Laboratory of Food Nutrition and Safety, Ministry of Education of China, Tianjin University of Science and Technology, Tianjin 300457, China

**Keywords:** acrylamide, detection, rapid methods, food safety

## Abstract

Acrylamide (AA) is produced by high-temperature processing of high carbohydrate foods, such as frying and baking, and has been proved to be carcinogenic. Because of its potential carcinogenicity, it is very important to detect the content of AA in foods. In this paper, the conventional instrumental analysis methods of AA in food and the new rapid immunoassay and sensor detection are reviewed, and the advantages and disadvantages of various analysis technologies are compared, in order to provide new ideas for the development of more efficient and practical analysis methods and detection equipment.

## 1. Introduction

Acrylamide (AA) is a small molecule organic compound that exists in solid form at normal temperature and pressure. It is sensitive to the light and can be initiated to polymerize to form polyacrylamide under ultraviolet conditions [1,2]. Therefore, AA is a commonly-used polymerization monomer in industry. In 1994, AA was classified as a “probable carcinogen” by the International Cancer Agency (IARC) and in April 2002, researchers demonstrated that plant foods rich in carbohydrates and low in protein are prone to produce large amounts of AA during high-temperature (>120 °C) processing such as frying and baking [3,4]. This result has caused widespread concern about this compound worldwide. The thermal processing of food is an indispensable process in modern food processing. Under heat treatment such as frying and baking, foods rich in starch and other carbohydrates have color, flavor, and other characteristics added through the Maillard reaction, which is the main way to form AA [5,6,7]. Recently, AA generation has been associated with high sterilization temperatures, mainly involving the formation of AA in fat-rich foods such as ripe black table olives [8,9]. The content of AA in high-carbohydrate foods with different thermal processing methods is different, and within a certain temperature range, the content of AA increases with the processing time and temperature [10,11,12,13]. According to the report of Commission Regulation (EU) 2017/2158 which established mitigation measures and benchmark levels for the reduction of the presence of acrylamide in food, the average AA content in food (processed cereal products, coffee substitutes, etc.) is in the range of 40–4000 µg kg^−1^ [14]. Since foods proposed to monitor the presence of AA in the Commission Regulation (EU) 2019/1888 (potato products, bakery products, cereal products, and others such as dried fruits, olives in brine) are an important part of human food, it is particularly important to deepen the research and quantitative analysis of the process control of AA content in foods [15,16,17].

In recent years, the mechanism of AA production and its mutagenesis and carcinogenesis in the human body have been gradually revealed [18,19,20,21] and related strategies for AA detection in various foods have been successively developed. These strategies are not only used for the analysis of AA content in foods, but also provide a reliable judgement of AA risk level [22]. It has been reported that the tolerable daily intake (TDI) of neurotoxic and carcinogenic AA is 40 and 2.6 μg kg^−1^ day^−1^, respectively [23]. On the other hand, the matrix of heat-processed foods rich in carbohydrates is usually complicated. In addition, AA has a small molecular weight (Mr = 71.08 g mol^−1^), high reactivity, and other characteristics, which makes it difficult to perform accurate quantitative analysis of AA. Therefore, it is of great significance to develop accurate, sensitive, and anti-interference methods for the analysis and detection of AA content in foods.

This paper reviews the conventional instrumental methods for AA detection in foods and new types of analytical methods such as rapid immunoassays, supramolecular recognition, and nano-biosensors, and comprehensively evaluates the advantages and the shortcomings of various analytical techniques, aiming to provide new ideas for the development of more-efficient and practical analytical methods and testing devices, so as to provide technical support for the detection and risk assessment of AA in foods.

## 2. Instrumental Analysis Strategies for AA Content in Foods

Up to now, instrumental analysis based on the principles of chromatography and mass spectrometry including high performance liquid chromatography (HPLC) [24,25,26], gas chromatography (GC) [27,28,29], liquid chromatography tandem mass spectrometry (LC-MS/MS) [30,31,32], and gas chromatography-mass spectrometry (GC-MS) [33] have still been the main methods to detect AA content in foods. With high accuracy and sensitivity, as well as good stability and reproducibility, these kinds of methods are the most reliable for analysis and detection of AA. Therefore, although these kinds of methods need expensive equipment and are high in detection cost, they are still the main methods for detecting AA content in food. Luo et al. have developed a non-aqueous reaction system based on the GC-MS method for rapid and sensitive detection of AA in food matrices [34]. Under mild reaction conditions (40 °C), concentrated AA can complete the reaction with flavanol in 1 min, which simplifies the derivatization reaction process and improves the stability of the detection results. Under optimal conditions, this developed GC-MS method has a linear response range of 0.005–4 μg mL^−1^ with correlation coefficient (R^2^) at 0.99993 in food matrices. The limit of detection (LOD, S/N = 3) and the relative standard deviation (RSD, *n* = 6) are achieved at 0.7 μg kg^−1^ and 2.3–6.1%, respectively, showing good accuracy, sensitivity, and repeatability, which can meet the needs of detection of AA in food matrix. However, due to the high polarity, low volatility, and low molecular weight of AA, the derivatization process is often needed to enhance the stability of AA, and further improve the detection sensitivity of GC and its combination technology. LC-MS/MS, however, has no derivatization process, greatly reducing the detection time and meeting the requirements for a green environment [35,36]. Calbiani and his co-workers established a fast and accurate method for the determination of AA in cooked food samples by reversed-phase LC-MS coupled with electrospray [37]. An acidified water extraction step without purification was used in this method, simplifying sample-processing procedures. Remarkable results (LOD: <15 μg kg^−1^; LOQ: <25 μg kg^−1^) were obtained for intraday repeatability (RSD < 1.5%) and between-day precision (RSD < 5%), demonstrating that this method is suitable for the determination of AA in cooked food products. Galuch et al. extracted AA from coffee samples by the method of dispersion liquid–liquid microextraction, combined with ultra-performance LC-MS/MS and standard addition method, obtaining good detection sensitivity (LOD: 0.9 μg L^−1^; limit of quantitation (LOQ): 3.0 μg L^−1^) and precision (internal and inter-assay precision: 6–9%) [38]. Tolgyesi developed a hydrophilic interaction liquid chromatography tandem mass spectrometric (HILIC-MS/MS) to determine AA in gingerbread samples with high sugar content [39]. The proposed method had acceptable accuracy (101–105%) and precision (2.9–7.6%) with a LOQ of 20 μg kg^−1^. At the same time, the method was also applied to other food samples (bread, roasted coffee, instant coffee, cappuccino powder, and fried potatoes), and the tested AA content was lower than the EU-set level. Additionally, because of the good separation effect, LC-MS/MS can also be applied in simultaneous detection of AA and other harmful substances in one sample, which has good application value [40,41]. Wu et al. used isotope-dilution ultra-performance LC-MS/MS for simultaneous detection of 4-methylimidazole and AA in 17 commercial biscuit products [42], revealing the wide presence of *4*-methylimidazole and AA in biscuit products. This method was validated with respect to linearity, LOQ, precision, trueness, and measurement uncertainty and offers a reliable and sensitive tool for *4*-MI and AA measurements in biscuit products.

**Figure 1:**
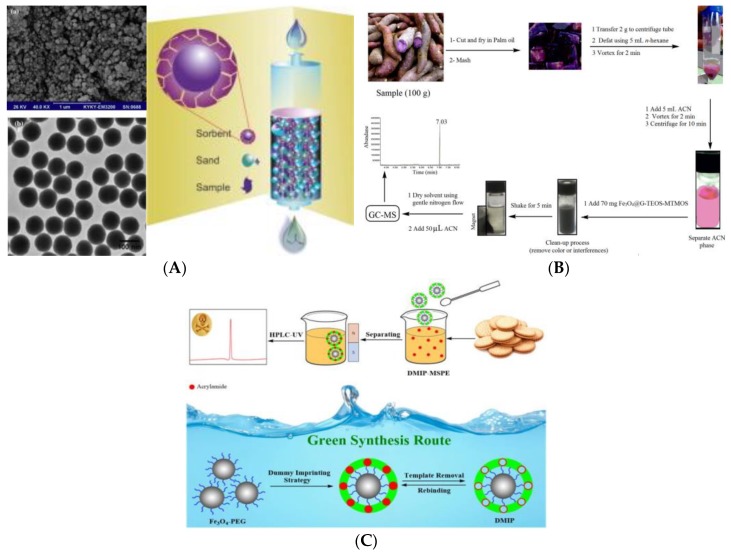
(**A**) Surface topography of dummy molecularly-imprinted silica nanoparticles (DMISNPs) (a: SEM and b: TEM) and the DMISNPs-matrix solid-phase dispersion method (MSPD) extraction procedure [45]. Copyright: Food Chemistry, 2016. (**B**) Schematic procedure of magnetic solid-phase extraction (MSPE) using Fe_3_O_4_@graphene-TEOS-MTMOS [49]. Copyright Food Chemistry, 2018; (**C**) Schematic procedure of MSPE using dummy molecularly-imprinted polymer (DMIP) with Fe_3_O_4_@PEG as core [50]. Copyright: Talanta, 2019.

In addition, because foods are complex matrices, analytical methods using large precision instruments often require a relatively tedious process for sample purification. Therefore, developing effective and reliable materials for sample pretreatment and purification is meaningful to improve sensitivity and accuracy of AA detection, and has very important application value [43,44]. Arabi and his co-workers have prepared dummy molecularly-imprinted silica nanoparticles (DMISNPs) with high selectivity for AA based on the techniques of sol-gel, one-step synthesis and central composite design [45]. In the polymerization process, *3*-aminopropyltrimethoxysilane (APTMS) was used as the functional monomer, propionamide as the dummy template, and tetraethyl orthosilicate (TEOS) as the crosslinking agent. The obtained DMISNPs were further used as sorbent to extract AA from food samples using a matrix solid-phase dispersion method (MSPD), and then combined with HPLC-MS to detect AA in biscuits and bread samples. The results showed that DMISNPs have high porosity, good uniformity and high selectivity and affinity for AA. More importantly, this molecularly-imprinted polymer (MIP) composite was easy to completely remove the dummy templates to obtain highly-recognized cavities, which are beneficial to eliminate template problems and improve mass transfer and extraction efficiency (Figure 1A). The MSPD method also greatly reduces the consumption of toxic organic solvents. Magnetic solid-phase extraction (MSPE) consumes less organic solvent and has higher contact-surface efficiency and repeatability. In addition, the magnetic adsorbent does not require the processes of filtration, centrifugation, and precipitation, and can be directly collected magnetically, which greatly simplifies the pretreatment steps and has received great attention in complex sample pretreatment techniques in recent years [46,47,48]. Nodeh successfully developed a hybrid of magnetite (Fe_3_O_4_) and sol-gel of TEOS and methyltrimethoxysilane (MTMOS) to modify the graphene. The obtained material was further applied as magnetic solid purified adsorbent for rapid purification and extraction of AA in various foods, combined with GC-MS analysis (Figure 1B) [49]. Compared with previous studies based on MSPE, this study used the matrix-matching method for calibration, which has better linearity (R^2^ = 0.9990), lower LOD (0.061–2.89 μg kg^−1^), and higher recovery (82.7–105.2%). The prepared Fe_3_O_4_@graphene-TEOS -MTMOS extractant can be reused at least seven times with a recovery rate higher than 85%. Bagheri et al. also used propionamide as a dummy template to fix a thin layer of chitosan-imprinting network on Fe_3_O_4_@PEG core in aqueous medium, and obtained a dummy MIP (DMIP) (Figure 1C), which was applied to detect AA in biscuit samples in combination with HPLC [50]. This DMIP had a uniform nano-core-shell structure and good magnetic properties, which were conducive to simple and rapid separation. This novel core-shell recognition material further overcame the shortcomings of poor selectivity of MSPE, and the synthesis was simple, easy to separate, in line with the green synthesis strategy, and very suitable for the pretreatment and purification of complex samples.

On the other hand, solid-phase microextraction (SPME) is a kind of non-solvent selective extraction method, which abandons the shortcomings of the traditional SPE process that needs column packing and solvent for desorption, and only needs a simple syringe to complete the whole pretreatment and injection processes. Therefore, SPME has the characteristics of low cost, simple device and operation, fast, efficient, and high sensitivity. As a unique sample pretreatment and enrichment method, SPME has also been paid attention to in the detection of AA [51,52]. A direct, fast strategy based on headspace SPME has been developed for AA extraction from coffee beans [53]. The commercial SPME fiber-coated polydimethylsiloxane (PDMS) was employed to carry out the silylation reaction of AA with *N,O*-bis(trimethylsilyl) trifluoroacetamide and further quantified AA analysis in combination with GC-MS methods. The LOQ of AA for this method is 3 μg kg^−1^ with good reproducibility (RSD: 2.6%), which was in accordance with the EU’s recommendations for monitoring AA content in foods [54]. The liquid-phase microextraction (LPME) method realizes the integration of sampling, separation, purification, concentration, and injection, which is simple and fast in AA detection [55,56]. Elahi et al. have developed a dispersive liquid microextraction combined with GC-MS method to detect AA in cookie samples [57]. This study has effectively removed the complex matrix components in sample pretreatment and significantly extracted trace amounts of target analytes in a short time. Lower values of LOD (0.6 μg kg^−1^) and LOQ (1.9 μg kg^−1^) and acceptable recovery range (89–95%) with RSD of 9.2% demonstrated the merits of the method in the detection of AA at low and high content in biscuits.

Stable isotope tracing technology is one technique that uses the enriched stable isotope-labeled compounds as tracers and analyzes isotopic compositions to monitor or detect certain biochemical processes [58,59]. At present, the main internal standard compounds used in the detection of AA by MS include d3-AA, ^13^C_3_-AA, *N, N*-dimethylacrylamide, propionic acid, and methacrylamide [60]. By adding ^13^C_3_-AA internal standard solution to the test sample, through a series of extraction, purification, and derivatization of bromine reagents, the GC-MS method can reach an LOD of 10 µg kg^−1^ of AA in rice [61]. Lim et al. employed the deuterated d3-AA as an internal standard for the analysis of AA content in food samples, and the established LC-MS/MS method achieved a lower LOD (0.04 μg kg^−1^) and LOQ (0.14 μg kg^−1^) [62]. The RSD values in the AA concentration range of 20–100 μg kg^−1^ was less than 8%, demonstrating good sensitivity and reproducibility of the developed method. This strategy did not require further extraction and purification processes, but still required a certain amount of toxic organic reagents. Ferrer-Aguirre et al. employed deuterated d5-AA as an internal standard, in combination with HPLC coupled to triple quadrupole-tandem MS, to initially determine AA content in different starchy foods (such as potato chips and potatoes) [63]. This effective analysis strategy used the water as an extraction solvent, which minimized the detection cost and reduced the sample processing. The values of LOD and LOQ were 4 and 12 μg kg^−1^ (potato chips) and 2 and 5 μg kg^−1^ (roasted asparagus), respectively. This method has the advantages of simple process, low cost, and no toxicity, and is suitable for preliminary identification of AA in different starchy foods. Carbon-labeled internal standards were also used for the detection of AA content in foods. Yoshioka Toshiaki et al. developed a supercritical fluid chromatography tandem mass spectrometry (SFC-MS/MS) technique using ^13^C_3_-AA as an internal standard for rapid quantitative analysis of AA in various beverage, cereal, and confectionery samples [64]. Compared with methods using hydrogen-labeled internal standards, this proposed method has extremely high accuracy and sensitivity, simplifies the detection steps, and can quickly quantify low-concentration analytes, which has a very important practical value.

## 3. New Strategies for AA Analysis

Food belongs to fast consumer goods, which require fast detection speeds and high throughput, which puts forward new requirements for food analysis and detection. Although the traditional instrumental analysis of AA in foods has obvious advantages in detection stability and accuracy, it needs a relatively cumbersome sample pretreatment process, which makes it far behind in real-time, online and large-number sample analysis. With the rise and in-depth development of technologies such as immunity, sensing, and chips, some simple, fast, low cost, and convenient analytical strategies have been proposed and applied to the detection of AA content in foods.

### 3.1. Capillary Electrophoresis

Capillary electrophoresis (CE) has the characteristics of fast analysis speed and high separation efficiency, and requires a small amount of sample, making it an effective tool for the analysis of trace components in foods [65,66]. CE is based on different charge ratios of the target substance to achieve efficient separation. Therefore, the target substance is required to have a certain charge (positive or negative). The non-charged AA can achieve the detection purpose by adding an ionic surfactant to the detection system to form a charged micelle on its surface. Abd El-Hady et al. developed an analyte focusing by ionic liquid micelle collapse (AFILMC) capillary electrophoresis method combined with ionic liquid ultrasonic-assisted extraction to simultaneously measure AA, asparagine, and glucose in foods [67]. In this process, *1*-butyl-*3*-methylimidazolium bromide (BMIM⁺ Br^−^) was used as a surfactant, and the washing procedure of HCl and water was appropriately optimized to sufficiently reduce the adsorption of BMIM⁺ Br^−^. The separation and extraction efficiency exceeded 97.0%. The AFILMC measurements achieved adequate reproducibility and accuracy with RSD 1.14–3.42% (*n* = 15) and recovery 98.0–110.0% within the concentration range of 0.05–10.0 μmol L^−1^. The LODs achieved to 0.71 μg kg^−1^ for AA, 1.06 μg kg^−1^ for asparagine, and 27.02 μg kg^−1^ for glucose, respectively, with linearity ranged between 2.2 and 1800 μg kg^−1^. This method has the characteristics of environmental protection, low cost, high efficiency, and high selectivity. Pre-column derivatization is another method used in CE to charge AA. 

**Figure 2:**
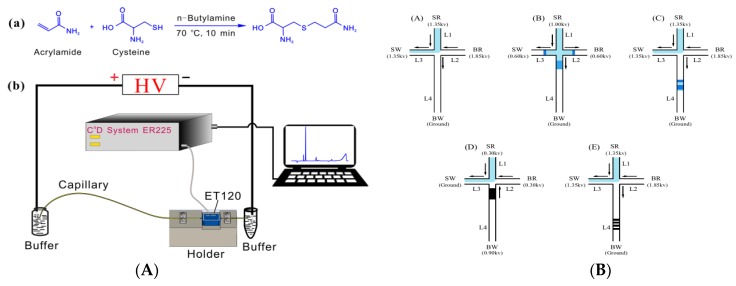
(**A**) Schematic illustration for thiol-ene click derivatization of acrylamide (AA) using cysteine and the CE-C^4^D system [68]. Copyright: Journal of Agricultural and Food Chemistry, 2019. (**B**) Five-steps of microchip electrophoresis technology (MCE) strategy. A: preloading, B: loading, C: prolonged field-amplified sample stacking, D: reversed-field stacking, and E: separation [69]. Copyright: Food Chemistry, 2016.

Yang et al. proposed an efficient method for AA derivatization based on thiol-olefin reaction using cysteine as a derivatization reagent, and combined with capacitively-coupled contactless conductivity detection (C^4^D) for CE analysis of AA (Figure 2A) [68]. This method can analyze labeled AA within 2.0 min, and the RSD of migration time and peak area are less than 0.84% and 5.6%, showing good accuracy and selectivity. At the same time, the C^4^D signal of the AA derivative has a good linear relationship with the AA concentration in the range of 7–200 μmol L^−1^ (R^2^ = 0.9991), LOD and LOQ (0.16 μmol L^−1^ and 0.52 μmol L^−1^). Due to the advantages of simple sample pretreatment, high derivatization efficiency, short analysis time, and high selectivity and sensitivity, this CE-C^4^D is expected to achieve further miniaturization for field analysis. 

A portable microchip requires a small amount of detection samples, especially when combined with electrophoresis technology, which shortens the separation channel, thus achieving faster separation and more sensitive detection [70,71]. Because the content of AA in foods is very low, it is not suitable for microchip electrophoresis technology (MCE). It must be combined with on-line enrichment technology to improve the sensitivity. This on-line enrichment and detection method effectively overcomes the interference of food complex matrix and improves the detection speed [72,73]. Wu et al. proposed an MCE based on a combination of high-field amplification and anti-field superposition of online multiple pre-enrichment technology for efficient analysis of AA in foods (Figure 2B) [69]. The best separation has been achieved under the condition of 100 mmol L^−1^ borate solution at pH 9.3 as the running buffer. The sensitivity of this method (LOD: 1 μg L^−1^) is 41–700 times higher than the previously-reported CE of on-line preconcentration technology, which has been successfully applied to detect AA content in potato chips and French fries with reliable results and satisfactory recoveries. Compared with traditional methods for AA detection, this effective method has the advantages of short analysis time, low sample and reagent consumption, and low instrumental cost.

### 3.2. Immunoassay Method

**Figure 3:**
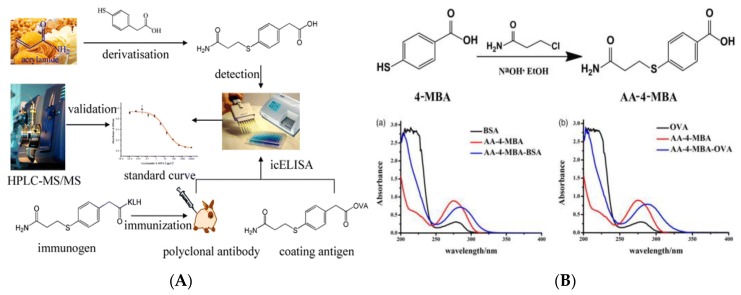
(**A**) The indirect competitive ELISA (ic-ELISA) procedure for AA analysis in foods [81]. Copyright: Journal of Agricultural and Food Chemistry, 2014. (**B**) Scheme for *4*-mercaptobenzoic acid-derived AA (AA-*4*-MBA) hapten synthesis and UV scans of curves of AA-*4*-MBA, protein, and Ag [82]. Copyright: Food and Agricultural Immunology, 2016.

Immunoassay is one new, rapid, and high-throughput analysis strategy based on the specific combination of antigen (Ag) and antibody (Ab). After nearly 20 years of development, the immunoassay has gradually developed into enzyme-linked immunoassay (ELISA) [74,75], chemiluminescent immunoassay [76,77], fluorescent immunoassay [78,79] and so on, which have been widely used in food analysis, especially in the field of rapid detection. The Ab with specific binding ability is the basis of immunoassay. Due to the fact that AA is a small molecular compound, lacking in antigenic determinant and immunogenicity, AA is usually cross-linked with the carrier proteins, bovine serum albumin (BSA), ovalbumin (OVA), etc., with immune response, to prepare incomplete antigens, and the polyclonal Abs with specific recognition ability are further obtained by immunizing animals. Singh et al. prepared polyclonal Abs which were raised against a hapten derived from AA and *3*-mercaptobenzoic acid (*3*-MBA) and established an indirect competitive ELISA (ic-ELISA) to quickly quantify AA in complex foods matrix and water [80]. This ic-ELISA had high affinity and specificity for AA-*3*-MBA derivatives and did not cross-react with the main precursors (asparagine, aspartic acid, AA, or *3*-MBA) that form AA in foods. The LODs achieved for AA-*3*-MBA in food matrices and water were 5.0 μg kg^−1^, and 0.1 μg L^−1^, respectively, which verified that the developed ic-ELISA has extremely high sensitivity and good AA recovery, and is suitable for AA detection in multiple matrices. Wu and his co-workers used the *4*-mercaptophenylacetic-acid-derived AA (AA-*4*-MPA) to prepare polyclonal Abs and developed a pre-analytical derivatization method for ic-ELISA analysis of AA (Figure 3A) [81]. By comparison with the results from the HPLC-MS/MS method, this ic-ELISA has better accuracy and reliability (IC_50_: 2.86 μg kg^−1^, LOD: 0.036 μg kg^−1^, linear range: 0.25–24.15 μg kg^−1^), lower detection cost, and is suitable for routine rapid screening of AA in food samples. Monoclonal antibodies (MAbs) are homologous Abs produced by a B-cell clone that recognize an antigenic determinant, have high titer, strong homogeneity and specificity, and low cross reactivity. Zhu et al. used the *4*-mercaptobenzoic acid-derived AA (AA-*4*-MBA) to couple to carrier proteins (BSA and OVA) (Figure 3B) [82]. The resulting conjugates of AA-*4*-MBA–BSA and AA-*4*-MBA–OVA were used as the immunogen and coating antigen. The obtained MAb is not specific for AA or *4*-MBA but has high affinity for AA-*4*-MBA (IC_50_: 32 μg L^−1^; LOD 8.87 μg L^−1^). The quantitative working range is 8.87–12.92 μg L^−1^ (IC_20_ to IC_80_) and the cross-reactivity with other analogs is less than 10%, meaning that the developed ic-ELISA method has extremely high specificity and can effectively detect AA in high-temperature-cooking foods.

Compared with the ELISA, the immunochromatographic strip (ICS) is a relatively mature integrated detection product, which is simpler to operate and can be completed without professional operators, meeting the real-time and fast-detection requirements of AA. Assaat et al. have produced, purified, and characterized a polyclonal Ab against AA for ICS testing of AA [83]. Polyclonal anti-AA Ab was prepared by injecting *N*-acryloxysuccinimide conjugated BSA hapten into New Zealand white rabbits and further purified with protein A and conjugated with Au nanoparticles (AuNPs). According to the obtained results, the ICS prepared in this study quantitatively showed that the intensity of the red line increased with the increase of AA concentration, and was sensitive to standard AA solution at 1 g L^−1^ concentration, which is expected to be applied for the rapid detection of AA in foods.

Immunoassay based on biological Abs has the advantage of fast detection speed and high throughput. However, the process of obtaining biological Abs with high specific binding ability is complicated and costly; it is easy to be affected by environmental conditions in the detection process, resulting in false positive results, which to some extent limit the development of biological immunoassay. Molecular imprinting technology (MIT) can chemically synthesize high-specific and stable polymers based on the principle of Ab formation. The obtained MIPs also called “artificial antibodies”, are used in the development of biomimetic ELISA method, which has very broad application prospects. A direct competitive biomimetic ELISA rapid analysis method for AA analysis was developed by Sun et al. using a hydrophilic-imprinted membrane as a biomimetic Ab [84]. In the preparing process of the imprinted membrane, -COOH of methacrylic acid reacted with -NH_2_ of AA, and an imprinted cavity and a specific binding site of -OH group were generated in a predetermined direction, so that the imprinted membrane had high binding and selectivity to AA. The developed biomimetic ELISA method had high sensitivity (IC_50_: 8.0 ± 0.4 mg L^−1^) and low LOD (IC_15_: 85.0 ± 4.2 μg L^−1^), and for an AA-blank potato sample, the recovery rate ranged from 90.0% to 111.5%. The biomimetic ELISA method is simple in pretreatment and does not require Ab coating and BSA/PBS blocking procedures, which greatly reduces the operation time. Additionally, the developed blotting membrane can be reused 20 times without loss of sensitivity, which greatly reduces the cost of analysis.

### 3.3. Sensor Analysis Technique

Sensors can on-line monitor the binding reaction between the tested substance and the recognition element, and convert the generated binding signal into a signal that can be processed quantitatively, such as electricity, light, or mass, to achieve the purpose of analysis and detection [85,86]. The AA molecule contains a -NH_2_ structure, which can be hydrolyzed to NH_4_^+^ and then detected by the selective electrode [87]. Because this method is based on the catalytic hydrolysis of -NH_2_, it has a strong cross-reaction to compounds containing -NH_2_ groups such as formamide and acetamide [88]. A new two-step waveform containing a process of separation of reverse-phase LC coupled to a pulsed amperometric detection was reported by Casella’s group for the quantification of low concentrations of AA in foodstuffs such as coffee and potato fries. Compared to the classical type of waveform, the proposed two-step waveform showed favorable analytical performance in terms of LOD (1.4 μg kg^−1^), precision, and improved long-term reproducibility.

#### 3.3.1. Electrochemical Sensing Analysis Based on Biomolecules 

As an emerging analysis strategy, biosensors have made in-depth developments in the fields of environment, medicine, and food. In food safety, various types of biosensors are designed for the analysis of food components and harmful substances [89,90,91,92]. Hemoglobin (Hb) is a redox-active protein that involves four polypeptide chains, each of which has an electroactive group of Fe^3+^/heme. The electrical activity of Hb is related to the reversible conversion of Hb-Fe^3+^ to Hb-Fe^2+^ [93,94]. At the same time, the valine α-NH_2_ in Hb can be combined with AA to form a complex, which causes the amount of Hb-Fe^2+^ to decrease, resulting in the change in the electron transfer on the surface of the sensing electrode. Based on this principle, the Hb can be modified on the surface of the transducer for AA detection [95]. Compared with other proteins with similar mechanisms of action (myoglobin, cytochrome c, etc.), Hb is more appropriate in the construction of a AA biosensor, due to its commercial accessibility at low cost, its relatively higher stability, and its configuration (*N*-(*2*-carbamoyl-ethyl)-*L*-valine), which is similar to that of the glycidamide (*N*-(*2*-carbamoyl-*2*-hydroxyethyl) -*RS*-valine), which facilitates the formation of Hb–AA adduct [96,97]. However, Hb has a complex spatial structure, and its electroactive center exists inside a polypeptide chain, which easily causes the electrode surface to be passivated and slows down the electron transfer rate. In recent years, in view of these shortcomings and problems, some meaningful solutions have been proposed. 

**Figure 4:**
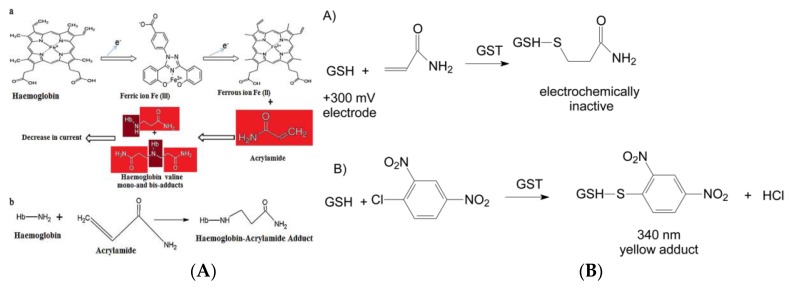
(**A**) a: Electrochemical reactions involved in the functioning of the hemoglobin nanoparticle (HbNP)-AA biosensor and b: adduct formation of HbNPs and AA. (**B**) Schematic representation of chemical reaction of the fabrication of HbNPs onto an Au electrode [98]. Copyright: International Journal of Biological Macromlecules, 2018.

**Figure 5:**
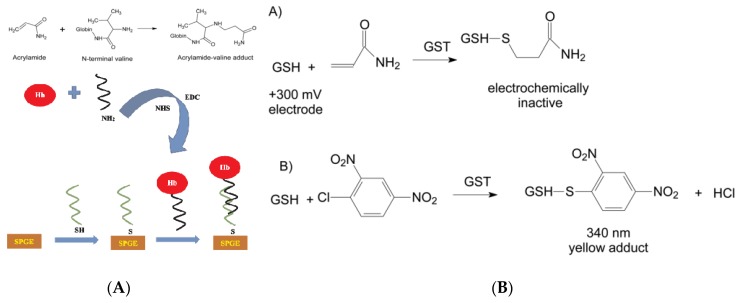
(**A**) Hb–AA adduct formation and schematic description of the preparation of dsDNA/Hb-modified screen-printed Au electrode for AA [99]. Copyright: Food Chemistry, 2019. (**B**) The reactions catalyzed by glutathione *S*-transferase (GST). A: coupling of GSH with AA and B: color reaction used for enzymatic activity measurements [100]. Copyright: Rsc Advances, 2018.

Yadav et al. prepared one kind of Hb nanoparticle (HbNP) by the desolvation method, and covalently immoblized HbNPs on a polycrystalline Au electrode to construct a current-type AA biosensor (Figure 4) [98]. At the experimental conditions of 20 °C, 0.26 V, and pH 5.0, the HbNP’s modified Au electrode showed the best current response within 2 s. In the water extract of foods at spiked AA concentration of 5 and 10 mmol L^−1^, a remarkable recovery of more than 95% was achieved, and the intra- and inter-assay coefficients of variation were lower than 5%. The wide-working range (0.1–100 nmol L^−1^) and the lower LOD (0.1 nmol L^−1^) signified this HbNP’s modified Au electrode could offer an effective measurement of AA in various processed foods. In this research, the use of HbNPs instead of natural Hb molecules solved the problem that Hb easily causes the electron transfer rate on the electrode surface to slow down, increases the specific surface area, and enables highly-sensitive micro detection of AA. In addition, the constructed sensor was not affected by the structural analogs of AA (such as acrylic acid and propionic acid) during the working process, signifying its good selective recognition ability. Asnaashari and his co-workers designed an effective double-stranded DNA (dsDNA)/Hb-modified screen-printed Au electrode for the detection of AA (Figure 5A) [99]. The square wave voltammetry (SWV) was used to monitor the current response of the designed biosensor to AA and AA-valine adduct, as well as the changes in the reduction/oxidation process of Hb-Fe^3+^/Hb-Fe^2+^. This fabricated sensor obtained a linear working range of AA (2.0 × 10^−6^ to 5.0 × 10^−2^ mol L^−1^) and a lower LOD (1.58 × 10^−7^ mol L^−1^) and had good reproducibility and high stability, which is suitable for direct determination of AA in foods.

Studies have shown that most of the AA ingested in the body is metabolized in the liver, except for a small part (<10%) which is excreted in the prototype with urine [101,102,103,104]. Under the action of enzymes, AA can combine with glutathione (GSH) to form thioglycolic acid compound, which is further converted into glycidomide (GA). In the case of low AA dose, about 50% of AA will be converted to GA, while in the case of high dose AA, most of AA will react with GSH and about 13% will be converted to GA. Therefore, GSH can also be modified on the electrode surface for the sensing response of AA in foods. Bucur et al. have proposed a method based on the amperometric monitoring of the coupling reaction between reduced glutathione (GSH) and AA catalyzed by glutathione *S*-transferase (GST) to produce an electrochemically-inactive compound (Figure 5B) [100]. Cobalt phthalocyanine was modified on a screen-printed electrode to monitor the decrease in GSH concentration at +300 mV, further aimed to detect the target AA. At the optimal GSH concentration (100 μmol L^−1^), the linear range for AA analysis was 7–50 μmol L^−1^ and LOD achieved to 5 μmol L^−1^. This proposed method was simple, did not require auxiliary substrates such as *1*-chloro-*2*,*4*-dinitrobenzene (CDNB), and did not need to suppress adverse competitive kinetics. The whole detection process was not affected by interfering compounds usually found in foods and could be applied for real sample analysis.

**Figure 6:**
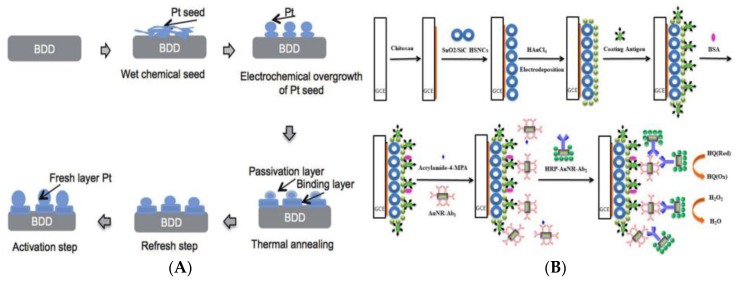
(**A**) Schematic representation of the Pt modification of a boron-doped diamond electrode (BDD) [105]. Copyright: Sensors and Materials, 2019. (**B**) Schematic illustration of the AA immunosensor fabrication [106]. Copyright: Analytica Chimica Acta, 2019.

Experimental factors such as the electron transfer rate on the electrochemical sensing interface, and the immobilization capacity and biological activity of the identification element have profoundly affected the performance of the sensors. The introduction of nanomaterials not only increases the electron transfer rate, but also increases the fixed amount and activity of biometric recognition elements, which can significantly improve the stability and sensitivity of sensors. Wulandar et al. developed a platinum (Pt) and Hb-modified boron-doped diamond electrode (Pt-BDD) for the construction of AA biosensors (Figure 6A) [105]. The surface of Pt-BDD modified with PtNPs had excellent stability. Meanwhile, Hb-Pt-modified BDD (Hb-Pt-BDD) showed a linear CV response in acetate buffered saline (0.2 mol L^−1^, pH 4.8) with AA concentration range of 0.01–1 nmol L^−1^. The LOD and LOQ achieved to 0.0085 nmol L^−1^ and 0.026 nmol L^−1^, respectively. These results demonstrated that the prepared Hb-Pt-BDD electrode has high stability, good sensitivity, and is reusable because it removes Hb adducts without removing Pt on the surface of BDD. Compared with PtNPs, Au nanomaterials also have excellent catalytic activity, efficient electron transfer performance, and good optical characteristics, and have been widely used in the sensing fields. Figure 6B shows the development of an ultrasensitive immunosensor using chitosan/SnO_2_-SiC hollow-sphere nanochains/AuNPs as signal amplification for detecting AA in water and food samples [106]. SnO_2_-SiC hollow-sphere nanochains with high surface area and AuNPs with good electrical conductivity were prepared on the surface of glassy carbon electrodes pre-coated with chitosan for subsequent fixed coating of antigens. Under this working mode, the constructed immunosensor has a lower LOD of 45.9 ± 2.7 ng kg^−1^ and wider working range of 187 ± 12.3 ng kg^−1^ to 104 ± 8.2 mg kg^−1^ at the optimized conditions. The recovery of AA in the spiked samples was in the range of 86.0–115.0%. The immunosensor exhibited a sensitive response to AA, and acceptable repeatability and stability. 

Carbon-based nanomaterials (carbon nanotubes, nanocarbon spheres, graphene, carbon nanofibers, etc.) have unique conductive and good mechanical properties, such as unique nanometer size, high surface area, high electron transfer rate, high stability and the ability to be modified on the surface that can maintain the stability and activity of biorecognition molecules [107,108]. Liu et al. have combined the composite of AuNPs-multi-walled carbon nanotubes (MWCNTs )-chitosan (AuNPs-MWCNTs-CS) with sol-gel MIT to construct a molecularly-imprinted electrochemical sensor for AA detection [109]. The composite of AuNPs and MWCNTs was introduced to improve the polymer conductivity and expand the surface area of electrode. At the working potential of 0–0.4 V, this developed electrochemical sensor exhibits a linear current response to AA in the concentration range of 0.05–5 mg L^−1^ with LOD of 0.028 mg L^−1^ (S/N = 3). With the characteristics of good repeatability, stable and reliable storage, good selectivity, high sensitivity, and low cost, this molecularly-imprinted electrochemical sensor has a very broad application prospect. 

**Figure 7:**
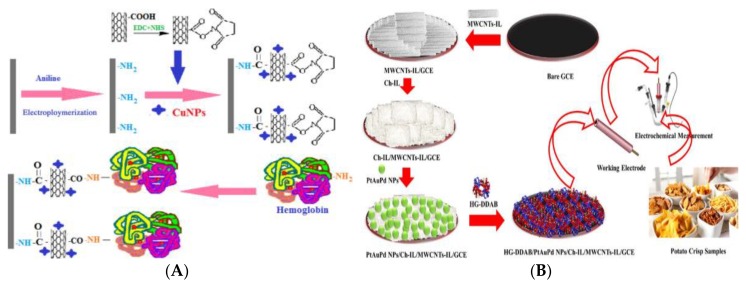
(**A**) Schematic representation of the fabrication of Hb/c-MWCNTs/CuNPs/PANI/PG [110]. Copyright: Analytical Biochemistry, 2013. (**B**) Schematic representation of the biosensor based on Hb-DDAB/PtAuPd NPs/Ch-IL/MWCNTs-IL/GCE [111]. Copyright: Talanta, 2018.

Carbon nanomaterials and their composites are also used in Hb-based AA electrochemical biosensors to enhance the surface electron transfer rate of electrodes. Batra et al. have developed an Hb electrochemical biosensor based on the composite of carboxylated MWCNTs/CuNPs/polyaniline (PANI) (c-MWCNTs/CuNPs/PANI), which can detect AA with high sensitivity (Figure 7A) [110]. Under the optimized experimental conditions, the fabricated sensor has low LOD (0.2 nmol L^−1^), high sensitivity (72.5 μA nmol L^−1^ cm^−2^), fast response time (<2 s), and wide linear range (5 nmol L^−1^ to 75 mmol L^−1^). When stored at 4 °C, the electrode can be used 120 times in 100 days with acceptable repeatability and stability. Varmiraa et al. also constructed an effective electrochemical biosensor based on Hb-dimethyldioctadecyl ammonium bromide (DDAB)/Pt-Au-palladium three metallic alloy NPs chitosan-*1*-/ethyl-*3*-methylimidazolium bis(trifluoromethylsulfonyl)imide/MWCNTs-IL/glassy carbon electrode (Hb-DDAB/PtAuPd NPs/Ch-IL/MWCNTs-IL/GCE) for selective and sensitive determination of AA in food samples (Figure 7B) [111]. The developed sensor can determine AA in two linear concentration ranges of 0.03–39.0 nmol L^−1^ and 39.0–150.0 nmol L^−1^ using SWV with LOD of 0.01 nmol L^−1^ and can selectively detect the target AA even in the presence of high concentrations of common interferences, confirming its highly selectivity. From the experimental results, it has been confirmed that the proposed sensor has a short response time (<8 s), good sensitivity, long-term stability, repeatability and reproducibility, and is capable of successfully measured AA in potato chips. 

In summary, electrochemical sensing methods for AA detection have certain selectivity, high sensitivity, good reproducibility, and cannot be interfered with by other ingredients in foods. This gives them a unique advantage in the analysis of AA content in foods. 

#### 3.3.2. Fluorescence Sensing Analysis Method

**Figure 8:**
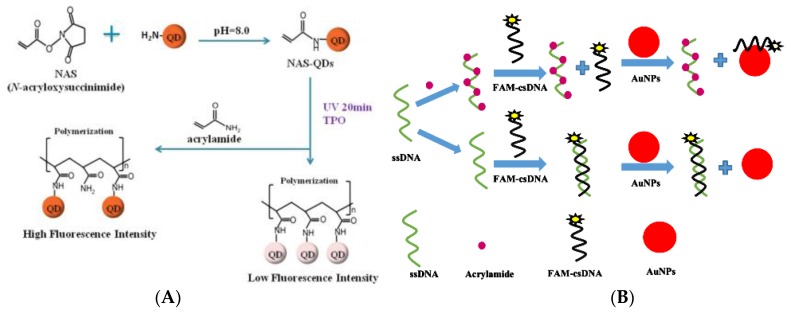
Schematic representation of fluorescent methods for AA detection. (**A**) CdSe/ZnS quantum dots (QDs) [122]. Copyright: Biosensors & Bioelectronics, 2014. (**B**) AuNPs and FAM-dsDNA [124]. Copyright: Sensors and Actuators B-Chemical, 2018.

Fluorescence sensors express signals generated by molecular recognition in the form of fluorescence (changes in fluorescence intensity and wavelength) to achieve information transmission [112,113]. Due to the merits of high sensitivity, good selectivity, and convenient use, fluorescence sensing analysis has been widely used and has made great progress in recent years [114,115,116]. Quantum dots (QDs) have unique photophysical properties such as high fluorescence quantum yield, size-controlled fluorescence, and photobleaching resistance, and have been widely used in the field of fluorescence sensing [117,118]. Since the AA molecule does not have fluorescent properties, it needs to be detected using other fluorescent substances. This kind of chemical reaction-based fluorescence sensing detection mode needs further research in terms of enhancing the detection sensitivity and selectivity. Especially when introducing new nanomaterials with unique properties into the fluorescence detection system, it is possible to develop more efficient and accurate analysis strategies [119,120,121]. Hu et al. have proposed a fluorescence sensing method for online detection of AA in potato chips, which was based on the increase of the distance between QDs caused by AA polymerization (Figure 8A) [122]. The UV light irradiation caused the C=C bond polymerization of *N*-acryloxysuccinimide-modified QDs, which shortened the distance between the QDs, leading to a decrease in fluorescence intensity. When AA is present in the tested sample, AA would participate in the above polymerization reaction, causing an increase of fluorescence intensity. The linear range and LOD of the established method reached 3.5 × 10^−5^–3.5 g L^−1^ (R^2^ = 0.94) and 3.5 × 10^−5^ g L^−1^, respectively. Although the sensitivity and specificity of this method cannot be compared with those of standard instrumental analysis, it greatly reduces the cost and time of detection and is suitable for the rapid detection of AA online in food processing. The ZnS QDs doped with Mn^2+^ were added onto graphene oxide as a fluorescent source to prepare an AA-MIP, which was successfully used as an environmentally-friendly fluorescent probe for AA detection [123]. When AA was adsorbed by AA-MIP, the fluorescence of ZnS QDs-doped was quenched, and the quenching effect was much stronger than that of non-imprinted polymers. The excitation and emission spectra of AA-MIP peaked at 325 nm and 601 nm, respectively. Under the optimal experimental conditions, the fluorescence of ZnS QDs in AA-MIP decreased within a linear range of 0.5–60 μmol L^−1^ AA concentration, and the LOD reached 0.17 μmol L^−1^. In the spiked water, acceptable recovery in a range of 100.2–104.5% with remarkable RSD of 1.9–3.9% was obtained, signifying the great accuracy and sensitivity of the developed methods. 

Because of their excellent optical and catalytic properties, easy synthesis, high chemical stability and selectivity, and high absorption coefficient, colloidal AuNPs are widely used as fluorescence quenchers in fluorescence sensors [125]. Figure 8B shows a simple, fast, and accurate fluorescence sensor using AuNPs and FAM-labeled dsDNA (FAM-dsDNA) for AA detection [124]. The detection principle was that the AA target present in the environment forms an adduct with single-stranded DNA, and the freely-existing FAM-labeled complementary strand DNA was adsorbed on the surface of AuNPs, causing the AuNPs quench. This proposed fluorescence sensor could be quickly assembled and complete the detection process in a short time, and have high sensitivity and selectivity, and wide linear response range of AA (1 × 10^−7^–0.05 mol L^−1^) and LOD of 1 × 10^−8^ mol L^−1^.

## 4. Conclusions

In recent years, various strategies based on different principles have been successively developed for the analysis of AA content in different food matrices. In contrast, the traditional instrumental analysis strategies based on chromatographic separation and mass spectrometry are still the first choice for AA analysis, due to the advantages of high accuracy and good reproducibility. In follow-up research, the development of efficient, stable, cheap, and convenient pretreatment purification materials for food matrices will continue to be one of the research hotspots. ELISA analysis kits and immunoassay test strips with the characteristics of high throughput and low cost have broad application prospects in rapid screening of large numbers of samples, but they need to be improved in terms of detection stability and environmental adaptability. Electrochemical and fluorescence sensing technologies need further research in the construction of sensing interfaces and the improvement of stability. The excellent electrical and optical properties of various nanomaterials provide new ideas for developing nano-sensing methods with high sensitivity, high throughput, and good reproducibility. 

## References

[1] Friedman M. (2003). Chemistry, biochemistry, and safety of acrylamide. A review. J. Agric. Food Chem..

[2] Altunay N., Gürkan R., Orhan U. (2016). A preconcentration method for indirect determination of acrylamide from chips, crackers and cereal-based baby foods using flame atomic absorption spectrometry. Talanta.

[3] IARC (1994). IARC Monographs on the Evaluation of Carcinogenic Risks to Humans.

[4] Tareke E., Rydberg P., Karlsson P., Eriksson S., Törnqvist M. (2002). Analysis of acrylamide, a carcinogen formed in heated foodstuffs. J. Agric. Food Chem..

[5] Mottram D.S., Wedzicha B.L., Dodson A.T. (2002). Acrylamide is formed in the maillard reaction. Nature.

[6] Stadler R.H., Varga N., Robert F., Hau J., Guy P.A., Robert M.C., Riediker S. (2002). Acrylamide from Maillard reaction products. Nature.

[7] Claeys W.L., De Vleeschouwer K., Hendrickx M.E. (2005). Kinetics of acrylamide formation and elimination during heating of an asparagine-sugar model system. J. Agric. Food Chem..

[8] Ehling S., Hengel M., Shibamoto T., Friedman M., Mottram D. (2005). Formation of acrylamide from lipids. Chemistry and Safety of Acrylamide in Food.

[9] Perez-Nevado F., Cabrera-Banegil M., Repilado E., Martillanes S., Martin-Vertedor D. (2018). Effect of different baking treatments on the acrylamide formation and phenolic compounds in Californian-style black olives. Food Control.

[10] Ehling S., Shibamoto T. (2005). Correlation of acrylamide generation in thermally processed model systems of asparagine and glucose with color formation, amounts of pyrazines formed, and antioxidative properties of extracts. J. Agric. Food Chem..

[11] Casado F.J., Montano A. (2008). Influence of processing conditions on acrylamide content in black ripe olives. J. Agric. Food Chem..

[12] Charoenprasert S., Mitchell A. (2014). Influence of California-style black ripe olive processing on the formation of acrylamide. J. Agric. Food Chem..

[13] Esposito F., Fasano E., De Vivo A., Velotto S., Sarghini F., Cirillo T. (2020). Processing effects on acrylamide content in roasted coffee production. Food Chem..

[14] Commission Recommendation (EU) 2017/2158 (2017). Commission Regulation (EU) 2017/2158 of 20 November 2017 establishing mitigation measures and benchmark levels for the reduction of the presence of acrylamide in food. Off. J. Eur. Union.

[15] Oracz J., Nebesny E., Zyzelewicz D. (2011). New trends in quantification of acrylamide in food products. Talanta.

[16] Hu Q.Q., Xu X.H., Fu Y.C., Li Y.B. (2015). Rapid methods for detecting acrylamide in thermally processed foods: A review. Food Control.

[17] Commission Recommendation (EU) 2019/1888 (2019). Commision Recommendation (EU) 2019/1888 of 7 November 2019 on the Monitoring of the Presence of Acrylamide in Certain Foods.

[18] Gokmen V., Palazoglu T.K. (2008). Acrylamide Formation in Foods during Thermal Processing with a Focus on Frying. Food Bioprocess Technol..

[19] El-Assouli S.M. (2009). Acrylamide in selected foods and genotoxicity of their Extracts. J. Egypt Public Health Assoc..

[20] Erkekoglu P., Baydar T. (2010). Toxicity of acrylamide and evaluation of its exposure in baby foods. Nutr. Res. Rev..

[21] Kumar J., Das S., Teoh S.L. (2018). Dietary acrylamide and the risks of developing cancer: Facts to ponder. Front. Nutr..

[22] Koszucka A., Nowak A., Nowak I., Motyl I. (2019). Acrylamide in human diet, its metabolism, toxicity, inactivation and the associated European Union legal regulations in food industry. Crit. Rev. Food Sci..

[23] Tardiff R.G., Gargas M.L., Kirman C.R., Carson M.L., Sweeney L.M. (2010). Estimation of safe dietary intake levels of acrylamide for humans. Food Chem. Toxicol..

[24] Singh P., Singh P., Raja R.B. (2010). Determination of acrylamide concentration in processed food products using normal phase high-performance liquid chromatography (HPLC). Afr. J. Biotechnol..

[25] Geng Z.M., Wang P., Liu A.M. (2011). Determination of acrylamide in starch-based foods by HPLC with pre-column ultraviolet derivatization. J. Chromatogr. Sci..

[26] Xu L.H., Zhang L.M., Qiao X.G., Xu Z.X., Song J.M. (2012). Determination of trace acrylamide in potato chip and bread crust based on SPE and HPLC. Chromatographia.

[27] Sun S.Y., Fang Y., Xia Y.M. (2012). A facile detection of acrylamide in starchy food by using a solid extraction-GC strategy. Food Control.

[28] Yao W.J. (2015). Direct determination of acrylamide in food by gas chromatography with nitrogen chemiluminescence detection. J. Sep. Sci..

[29] Saraji M., Javadian S. (2019). Single-drop microextraction combined with gas chromatography-electron capture detection for the determination of acrylamide in food samples. Food Chem..

[30] Zhang Y., Ren Y., Jiao J., Li D., Zhang Y. (2011). Ultra high-performance liquid chromatography-tandem mass spectrometry for the simultaneous analysis of asparagine, sugars, and acrylamide in Maillard reactions. Anal. Chem..

[31] De Paola E.L., Montevecchi G., Masino F., Garbini D., Barbanera M., Antonelli A. (2017). Determination of acrylamide in dried fruits and edible seeds using QuEChERS extraction and LC separation with MS detection. Food Chem..

[32] Fernández A., Talaverano M.I., Pérez-Nevado F., Boselli E., Cordeiro A.M., Martillanes S., Foligni R., Martín-Vertedor D. (2020). Evaluation of phenolics and acrylamide and their bioavailability in high hydrostatic pressure treated and fried table olives. J. Food Process Pres..

[33] Cagliero C., Ho T.D., Zhang C., Bicchi C., Anderson J.L. (2016). Determination of acrylamide in brewed coffee and coffee powder using polymeric ionic liquid-based sorbent coatings in solid-phase microextraction coupled to gas chromatography-mass spectrometry. J. Chromatogr. A.

[34] Luo L., Ren Y., Liu J., Wen X.D. (2016). Investigation of a rapid and sensitive non-aqueous reaction system for the determination of acrylamide in processed foods by gas chromatography-mass spectrometry. Anal. Methods.

[35] Jozinovic A., Sarkanj B., Ackar D., Balentic J.P., Subaric D., Cvetkovic T., Ranilovic J., Guberac S., Babic J. (2019). Simultaneous determination of acrylamide and hydroxymethylfurfural in extruded products by LC-MS/MS method. Molecules.

[36] Fernandes C.L., Carvalho D.O., Guido L.F. (2019). Determination of acrylamide in biscuits by high-resolution orbitrap mass spectrometry: A novel application. Foods.

[37] Calbiani F., Careri M., Elviri L., Mangia A., Zagnoni I. (2004). Development and single-laboratory validation of a reversed-phase liquid chromatography-electrospray-tandem mass spectrometry method for identification and determination of acrylamide in foods. J. AOAC Int..

[38] Galuch M.B., Magon T.F.S., Silveira R., Nicacio A.E., Pizzo J.S., Bonafe E.G., Maldaner L., Santos O.O., Visentainer J.V. (2019). Determination of acrylamide in brewed coffee by dispersive liquid-liquid microextraction (DLLME) and ultra-performance liquid chromatography tandem mass spectrometry (UPLC-MS/MS). Food Chem..

[39] Tolgyesi A., Sharma V.K. (2020). Determination of acrylamide in gingerbread and other food samples by HILIC-MS/MS: A dilute-and-shoot method. J. Chromatogr. B.

[40] Xiong J., Qian S., Xie Y.H., Xie Z.W., Li H.X. (2014). Simultaneous determination of acrylamide, aniline and benzidine in water sample by high performance liquid chromatography-tandem mass spectrometry. Chin. J. Anal. Chem..

[41] Lee K.J., Lee G.H., Kim H.S., Oh M.S., Chu S., Hwang I.J., Lee J.Y., Choi A., Kim C.I., Park H.M. (2015). Determination of heterocyclic amines and acrylamide in agricultural products with liquid chromatography-tandem mass spectrometry. Toxicol. Res..

[42] Wu C.J., Wang L., Guo X.B., Li H., Yu S.J. (2019). Simultaneous detection of 4(5)-methylimidazole and acrylamide in biscuit products by isotope-dilution UPLC-MS/MS. Food Control.

[43] Pugajeva I., Jaunbergs J., Bartkevics V. (2015). Development of a sensitive method for the determination of acrylamide in coffee using high-performance liquid chromatography coupled to a hybrid quadrupole orbitrap mass spectrometer. Food Addit. Contam. A.

[44] Omar M.M.A., Elbashir A.A., Schmitz O.J. (2015). Determination of acrylamide in Sudanese food by high performance liquid chromatography coupled with LTQ Orbitrap mass spectrometry. Food Chem..

[45] Arabi M., Ghaedi M., Ostovan A. (2016). Development of dummy molecularly imprinted based on functionalized silica nanoparticles for determination of acrylamide in processed food by matrix solid phase dispersion. Food Chem..

[46] Chang T.T., Yan X.Y., Liu S.M., Liu Y.X. (2017). Magnetic dummy template silica sol-gel molecularly imprinted polymer nanospheres as magnetic solid-phase extraction material for the selective and sensitive determination of bisphenol A in plastic bottled beverages. Food Anal. Method.

[47] Nasir A.N.M., Yahaya N., Zain N.N.M., Lim V., Kamaruzaman S., Saad B., Nishiyama N., Yoshida N., Hirota Y. (2019). Thiol-functionalized magnetic carbon nanotubes for magnetic micro-solid phase extraction of sulfonamide antibiotics from milks and commercial chicken meat products. Food Chem..

[48] Liu J.M., Lv S.W., Yuan X.Y., Liu H.L., Wang S. (2019). Facile construction of magnetic core-shell covalent organic frameworks as efficient solid-phase extraction adsorbents for highly sensitive determination of sulfonamide residues against complex food sample matrices. RSC Adv..

[49] Nodeh H.R., Ibrahim W.A.W., Kamboh M.A., Sanagi M.M. (2018). Magnetic graphene sol-gel hybrid as clean-up adsorbent for acrylamide analysis in food samples prior to GC-MS. Food Chem..

[50] Bagheri A.R., Arabi M., Ghaedi M., Ostovan A., Wang X.Y., Li J.H., Chen L.X. (2019). Dummy molecularly imprinted polymers based on a green synthesis strategy for magnetic solid-phase extraction of acrylamide in food samples. Talanta.

[51] Pourmand E., Ghaemi E., Alizadeh N. (2017). Determination of acrylamide in potato-based foods using headspace solid-phase microextraction based on nanostructured polypyrrole fiber coupled with ion mobility spectrometry: A heat treatment study. Anal. Method.

[52] Nematollahi A., Kamankesh M., Hosseini H., Ghasemi J., Hosseini-Esfahani F., Mohammadi A. (2019). Investigation and determination of acrylamide in the main group of cereal products using advanced microextraction method coupled with gas chromatography-mass spectrometry. J. Cereal. Sci..

[53] Wawrzyniak R., Jasiewicz B. (2019). Straightforward and rapid determination of acrylamide in coffee beans by means of HS-SPME/GC-MS. Food Chem..

[54] Commission Recommendation (EU) (2013). 2013/647 of 8 November 2013 on investigations into the levels of acrylamide in food. Off. J. Eur. Union.

[55] Zokaei M., Abedi A.S., Kamankesh M., Shojaee-Aliababadi S., Mohammadi A. (2017). Ultrasonic-assisted extraction and dispersive liquid-liquid microextraction combined with gas chromatography-mass spectrometry as an efficient and sensitive method for determining of acrylamide in potato chips samples. Food Chem..

[56] Faraji M., Hamdamali M., Aryanasab F., Shabanian M. (2018). 2-Naphthalenthiol derivatization followed by dispersive liquid-liquid microextraction as an efficient and sensitive method for determination of acrylamide in bread and biscuit samples using high-performance liquid chromatography. J. Chromatogr. A.

[57] Elahi M., Kamankesh M., Mohammadi A., Jazaeri S. (2019). Acrylamide in cookie samples: Analysis using an efficient co-derivatization coupled with sensitive microextraction method followed by gas chromatography-mass spectrometry. Food Anal. Method.

[58] Lim H.H., Shin H.S. (2013). Ultra trace level determinations of acrylamide in surface and drinking water by GC-MS after derivatization with xanthydrol. J. Sep. Sci..

[59] Backe W.J., Yingling V., Johnson T. (2014). The determination of acrylamide in environmental and drinking waters by large-volume injection-hydrophilic-interaction liquid chromatography and tandem mass spectrometry. J. Chromatogr. A.

[60] Wang H., Lee A.W., Shuang S., Choi M.M. (2008). SPE/HPLC/UV studies on acrylamide in deep-fried flour-based indigenous Chinese foods. Microchem. J..

[61] GB/T 5009 (2015). 204–2014. Determination of Acrylamide in Food.

[62] Lim H.H., Shin H.S. (2014). A new derivatization approach with d-cysteine for the sensitive and simple analysis of acrylamide in foods by liquid chromatography-tandem mass spectrometry. J. Chromatogr. A.

[63] Ferrer-Aguirre A., Romero-Gonzalez R., Vidal J.L.M., Frenich A.G. (2016). Simple and fast determination of acrylamide and metabolites in potato chips and grilled asparagus by liquid chromatography coupled to mass spectrometry. Food Anal. Method.

[64] Yoshioka T., Izumi Y., Nagatomi Y., Miyamoto Y., Suzuki K., Bamba T. (2019). A highly sensitive determination method for acrylamide in beverages, grains, and confectioneries by supercritical fluid chromatography tandem mass spectrometry. Food Chem..

[65] Başkan S., Erim F.B. (2007). NACE for the analysis of acrylamide in food. Electrophoresis.

[66] Voeten R.L.C., Ventouri I.K., Haselberg R., Somsen G.W. (2018). Capillary electrophoresis: Trends and recent advances. Anal. Chem..

[67] Abd El-Hady D., Albishri H.M. (2015). Simultaneous determination of acrylamide, asparagine and glucose in food using short chain methyl imidazolium ionic liquid based ultrasonic assisted extraction coupled with analyte focusing by ionic liquid micelle collapse capillary electrophoresis. Food Chem..

[68] Yang S.P., Li Y.T., Li F., Yang Z.Y., Quan F.F., Zhou L., Pu Q.S. (2019). Thiol-ene click derivatization for the determination of acrylamide in potato products by capillary electrophoresis with capacitively coupled contactless conductivity detection. J. Agric. Food Chem..

[69] Wu M.L., Chen W.J., Wang G., He P.G., Wang Q.J. (2016). Analysis of acrylamide in food products by microchip electrophoresis with on-line multiple-preconcentration techniques. Food Chem..

[70] Kitagawa F., Kawai T., Sueyoshi K., Otsuka K. (2012). Recent progress of on-line sample preconcentration techniques in microchip electrophoresis. Anal. Sci..

[71] Arvanitoyannis I.S., Dionisopoulou N. (2014). Acrylamide: Formation, occurrence in food products, detection methods, and legislation. Crit. Rev. Food Sci..

[72] Sueyoshi K., Kitagawa F., Otsuka K. (2013). Effect of a low-conductivity zone on field-amplified sample stacking in microchip micellar electrokinetic chromatography. Anal. Sci..

[73] Breadmore M.C., Tubaon R.M., Shallan A.I., Phung S.C., Keyon A.S.A., Gstoettenmayr D., Prapatpong P. (2015). Recent advances in enhancing the sensitivity of electrophoresis and electrochromatography in capillaries and microchips (2012–2014). Electrophoresis.

[74] Cui Y.L., Zhao J., Zhou J., Tan G.Y., Zhao Q.Y., Zhang Y.H., Wang B.M., Jiao B.N. (2019). Development of a sensitive monoclonal antibody-based indirect competitive enzyme-linked immunosorbent assay for analysing nobiletin in citrus and herb samples. Food Chem..

[75] Chen Y.J., Zhang S.P., Hong Z.S., Lin Y.Y., Dai H. (2019). A mimotope peptide-based dual-signal readout competitive enzyme-linked immunoassay for non-toxic detection of zearalenone. J. Mater. Chem. B.

[76] Zhou X.C., Shi J., Zhang J., Zhao K., Deng A.P., Li J.G. (2019). Multiple signal amplification chemiluminescence immunoassay for chloramphenicol using functionalized SiO_2_ nanoparticles as probes and resin beads as carriers. Spectrochim. Acta. A.

[77] Li J., Zhao X., Chen L.J., Qian H.L., Wang W.L., Yang C., Yan X.P. (2019). p-Bromophenol-enhanced bienzymatic chemiluminescence competitive immunoassay for ultrasensitive determination of aflatoxin B-1. Anal. Chem..

[78] Chen E.J., Xu Y., Ma B., Cui H.F., Sun C.X., Zhang M.Z. (2019). Carboxyl-functionalized, europium nanoparticle-based fluorescent immunochromatographic assay for sensitive detection of citrinin in monascus fermented food. Toxins.

[79] Xiong Y., Zhang K.K., Gao B., Wu Y.Q., Huang X.L., Lai W.H., Xiong Y.H., Liu Y. (2019). Fluorescence immunoassay through histone-ds-poly(AT)-templated copper nanoparticles as signal transductors for the sensitive detection of salmonella choleraesuis in milk. J. Dairy Sci..

[80] Singh G., Brady B., Koerner T., Becalski A., Zhao T., Feng S., Godefroy S.B., Huet A.C., Delahaut P. (2014). Development of a highly sensitive competitive indirect enzyme-linked immunosorbent assay for detection of acrylamide in foods and water. Food Anal. Method.

[81] Wu J., Shen Y.D., Lei H.T., Sun Y.M., Yang J.Y., Xiao Z.L., Wang H., Xu Z.L. (2014). Hapten synthesis and development of a competitive indirect enzyme-linked immunosorbent assay for acrylamide in food samples. J. Agr. Food Chem..

[82] Zhu Y.T., Song S.S., Liu L.Q., Kuang H., Xu C.L. (2016). An indirect competitive enzyme-linked immunosorbent assay for acrylamide detection based on a monoclonal antibody. Food Agric. Immunol..

[83] Assaat L.D., Saepudin E., Soejoedono R.D., Adji R.S., Poetri O.N., Ivandini T.A. (2019). Production of a polyclonal antibody against acrylamide for immunochromatographic detection of acrylamide using strip tests. J. Anim. Vet. Adv..

[84] Sun Q., Xu L.H., Ma Y., Qiao X.G., Xu Z.X. (2014). Study on a biomimetic enzyme-linked immunosorbent assay method for rapid determination of trace acrylamide in French fries and cracker samples. J. Sci. Food Agric..

[85] Sarkar D., Xie X.J., Anselmo A.C., Mitragotri S., Banerjee K. (2014). MoS_2_ Field-effect transistor for next-generation label-free biosensors. ACS Nano..

[86] Hu Q.Q., Wang R.H., Wang H., Slavik M.F., Li Y.B. (2018). Selection of acrylamide-specific aptamers by a quartz crystal microbalance combined SELEX method and their application in rapid and specific detection of acrylamide. Sens. Actuators B Chem..

[87] Bhadani S.N., Prasad Y.K., Kundu S. (1980). Electrochemical and chemical polymerization of acrylamide. J. Polym. Sci. Pol. Chem..

[88] Casella I.G., Pierri M., Contursi M. (2006). Determination of acrylamide and acrylic acid by isocratic liquid chromatography with pulsed electrochemical detection. J. Chromatogr. A.

[89] Trojanowicz M., Hitchman M.L. (2015). Determination of pesticides using electrochemical biosensors. Trac-Trends Anal. Chem..

[90] Wu D., Du D., Lin Y. (2016). Recent progress on nanomaterial-based biosensors for veterinary drug residues in animal-derived food. Trends Anal. Chem..

[91] Pandey C.M., Malhotra B.D. (2018). Nanomaterials for Biosensors.

[92] Rasooly A., Herold K.E. (2019). Biosensors for the analysis of food-and waterborne pathogens and their toxins. J. Aoac. Int..

[93] Shiddiky M.J.A., Torriero A.A.J. (2011). Application of ionic liquids in electrochemical sensing systems. Biosens. Bioelectron..

[94] Batra B., Lata S., Pundir C.S. (2013). Construction of an improved amperometric acrylamide biosensor based on hemoglobin immobilized onto carboxylated multi-walled carbon nanotubes/iron oxide nanoparticles/chitosan composite film. Bioproc. Biosyst. Eng..

[95] Garabagiu S., Mihailescu G. (2011). Simple hemoglobin-gold nanoparticles modified electrode for the amperometric detection of acrylamide. J. Electroanal. Chem..

[96] Sayyad A.S., Balakrishnan K., Ci L., Kabbani A.T., Vajtai R., Ajayan P.M. (2012). Synthesis of iron nanoparticles from hemoglobin and myoglobin. Nanotechnology.

[97] Wu H., Wang X., Qiao M. (2015). Enhancing sensitivity of hemoglobin-based electrochemical biosensor by using protein conformational intermediate. Sens. Actuators B-Chem..

[98] Yadav N., Chhillar A.K., Pundir C.S. (2018). Preparation, characterization and application of haemoglobin nanoparticles for detection of acrylamide in processed foods. Int. J. Biol. Macromol..

[99] Asnaashari M., Kenari R.E., Farahmandfar R., Abnous K., Taghdisi S.M. (2019). An electrochemical biosensor based on hemoglobin-oligonucleotides-modified electrode for detection of acrylamide in potato fries. Food Chem..

[100] Bucur M.P., Bucur B., Radu G.L. (2018). Simple, selective and fast detection of acrylamide based on glutathione S-transferase. RSC Adv..

[101] Sumner S.C., MacNeela J.P., Fennell T.R. (1992). Characterization and quantitation of urinary metabolites of [1,2,3-^13^C]acrylamide in rats and mice using ^13^C nuclear magnetic resonance spectroscopy. Chem. Res. Toxicol..

[102] Hartmann E.C., Bolt H.M., Drexler H., Angerer J. (2009). *N*-Acetyl-S-(1-carbamoyl-2-hydroxy-ethyl)-l-cysteine (iso-GAMA) a further product of human metabolism of acrylamide: Comparison with the simultaneously excreted other mercaptuic acids. Arch. Toxicol..

[103] Hartmann E.C., Latzin J.M., Schindler B.K., Koch H.M., Angerer J. (2011). Excretion of 2,3-dihydroxy- propionamide (OH-PA), the hydrolysis product of glycidamide, in human urine after single oral dose of deuterium-labeled acrylamide. Arch. Toxicol..

[104] Luo Y.S., Long T.Y., Shen L.C., Huang S.L., Chiang S.Y., Wu K.Y. (2015). Synthesis, characterization and analysis of the acrylamide- and glycidamide-glutathione conjugates. Chem. Biol. Interact..

[105] Wulandari R., Ivandini T.A., Irkham, Saepudin E., Einaga Y. (2019). Modification of boron-doped diamond electrodes with platinum to increase the stability and sensitivity of haemoglobin-based acrylamide sensors. Sens. Mater..

[106] Wu M.F., Wang Y., Li S., Dong X.X., Yang J.Y., Shen Y.D., Wang H., Sun Y.M., Lei H.T., Xu Z.L. (2019). Ultrasensitive immunosensor for acrylamide based on chitosan/SnO_2_-SiC hollow sphere nanochains/gold nanomaterial as signal amplification. Anal. Chim. Acta.

[107] Rivas G.A., Rubianes M.D., Rodriguez M.C., Ferreyra N.E., Luque G.L., Pedano M.L., Miscoria S.A., Parrado C. (2007). Carbon nanotubes for electrochemical biosensing. Talanta.

[108] Deshmukh M.A., Jeon J.Y., Ha T.J. (2020). Carbon nanotubes: An effective platform for biomedical electronics. Biosens. Bioelectron..

[109] Liu X., Mao L.G., Wang Y.L., Shi X.B., Liu Y., Yang Y., He Z. (2016). Electrochemical sensor based on imprinted sol-gel polymer on Au NPs-MWCNTs-CS modified electrode for the determination of acrylamide. Food Anal. Method..

[110] Batra B., Lata S., Sharma M., Pundir C.S. (2013). An acrylamide biosensor based on immobilization of hemoglobin onto multiwalled carbon nanotube/copper nanoparticles/polyaniline hybrid film. Anal. Biochem..

[111] Varmira K., Abdi O., Gholivand M.B., Goicoechea H.C., Jalalvand A.R. (2018). Intellectual modifying a bare glassy carbon electrode to fabricate a novel and ultrasensitive electrochemical biosensor: Application to determination of acrylamide in food samples. Talanta.

[112] Wolfbeis O.S. (2005). Materials for fluorescence-based optical chemical sensors. J. Mater. Chem..

[113] Dandin M., Abshire P., Smela E. (2007). Optical filtering technologies for integrated fluorescence sensors. Lab Chip.

[114] Dhenadhayalan N., Lee H.L., Yadav K., Lin K.C., Lin Y.T., Chang A.H.H. (2016). Silicon quantum dots based fluorescence turn-on metal ion sensors in live cells. ACS Appl. Mater. Inter..

[115] Li X., Li Z., Yang Y.W. (2018). Tetraphenylethylene-interweaving conjugated macrocycle polymer materials as two-photon fluorescence sensors for metal ions and organic molecules. Adv. Mater..

[116] Al-Zahrani F.A.M., El-Shishtawy R.M., Asiri A.M., Al-Soliemy A.M., Abu Mellah K., Ahmed N.S.E., Jedidi A. (2020). A new phenothiazine-based selective visual and fluorescent sensor for cyanide. BMC Chem..

[117] Kong Y.L., Cheng Q., He Y., Ge Y.L., Zhou J.G., Song G.W. (2020). A dual-modal fluorometric and colorimetric nanoprobe based on graphitic carbon nitrite quantum dots and Fe(II)-bathophenanthroline complex for detection of nitrite in sausage and water. Food Chem..

[118] Huang B.H., Shen S.S., Wei N., Guo X.F., Wang H. (2020). Fluorescence biosensor based on silicon quantum dots and 5,5’-dithiobis-(2-nitrobenzoic acid) for thiols in living cells. Spectrochim. Acta A.

[119] Zhou Y., Ma Z.F. (2016). A novel fluorescence enhanced route to detect copper(II) by click chemistry-catalyzed connection of Au@SiO_2_ and carbon dots. Sens. Actuators B-Chem..

[120] Mani N.P., Ganiga M., Cyriac J. (2018). MoS_2_ nanohybrid as a fluorescence sensor for highly selective detection of dopamine. Analyst.

[121] Peng D., Zhang L., Liang R.P., Qiu J.D. (2018). Rapid detection of mercury ions based on nitrogen doped graphene quantum dots accelerating formation of manganese porphyrin. ACS Sens..

[122] Hu Q.Q., Xu X.H., Li Z.M., Zhang Y., Wang J.P., Fu Y.C., Li Y.B. (2014). Detection of acrylamide in potato chips using a fluorescent sensing method based on acrylamide polymerization-induced distance increase between quantum dots. Biosens. Bioelectron..

[123] Liu Y., Hu X., Bai L., Jiang Y.H., Qiu J., Meng M.J., Liu Z.C., Ni L. (2018). A molecularly imprinted polymer placed on the surface of graphene oxide and doped with Mn(II)-doped ZnS quantum dots for selective fluorometric determination of acrylamide. Microchim. Acta.

[124] Asnaashari M., Kenari R.E., Farahmandfar R., Taghdisi S.M., Abnous K. (2018). Fluorescence quenching biosensor for acrylamide detection in food products based on double-stranded DNA and gold nanoparticles. Sens. Actuators B-Chem..

[125] Amit N., Anupam B., Shweta P. (2016). Gold nanoparticle induced enhancement of molecular fluorescence for Zn2+ detection in aqueous niosome solution. Proceedings of the 13th International Conference on Fibre Optics & Photonics.

